# Supplementary material for: Predicting visual function by interpreting a neuronal wiring diagram
Source: Nature. 2024 Oct 2;634(8032):113–23. doi: 10.1038/s41586-024-07953-5 (PMC11446822; doi:10.1038/s41586-024-07953-5)
Supplement: Supplementary file 6 — CRF and ERF predictions for individual TmY4 and TmY9 cells. Analogous to Supplementary Data 3, but for TmY target types. Shown are the top four monosynaptic pathways, the strongest pathway passing through each of the top ten intermediary types (ranking from Extended Data Fig. 7), and the trisynaptic pathway Tm1–TmY–Dm3–TmY (see the section entitled Prediction of spatial normalization). [file 41586_2024_7953_MOESM6_ESM.zip › DataS4/TmY9qΓèÑ/720575940611112260.pdf]

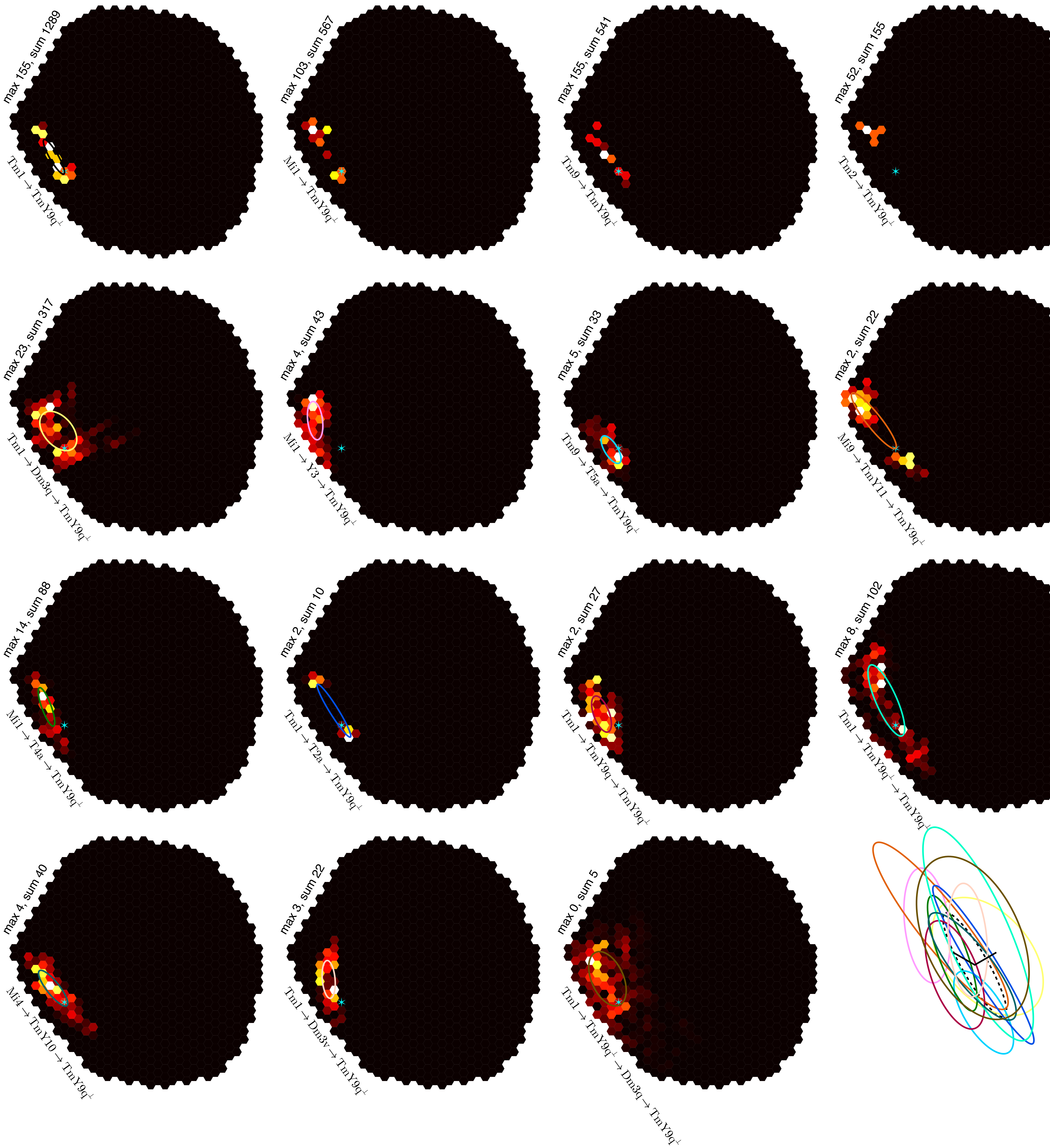
max 155, sum 1289
Tm1 → TmY9q⊥
max 103, sum 567
Mi1 → TmY9q⊥
max 155, sum 541
Tm9 → TmY9q⊥
max 52, sum 155
Tm2 → TmY9q⊥
max 23, sum 317
Tm1 → Dm3q → TmY9q⊥
max 4, sum 43
Mi1 → Y3 → TmY9q⊥
max 5, sum 33
Tm9 → T5a → TmY9q⊥
max 2, sum 22
Mi9 → TmY11 → TmY9q⊥
max 14, sum 88
Mi1 → T4a → TmY9q⊥
max 2, sum 10
Tm1 → T2a → TmY9q⊥
max 2, sum 27
Tm1 → TmY9q → TmY9q⊥
max 8, sum 102
Tm1 → TmY9q⊥ → TmY9q⊥
max 4, sum 40
Mi4 → TmY10 → TmY9q⊥
max 3, sum 22
Tm1 → Dm3v → TmY9q⊥
max 0, sum 5
Tm1 → TmY9q⊥ → Dm3q → TmY9q⊥
